# Comparative Study of Nanoparticle Blood Circulation after Forced Clearance of Own Erythrocytes (Mononuclear Phagocyte System-Cytoblockade) or Administration of Cytotoxic Doxorubicin- or Clodronate-Loaded Liposomes

**DOI:** 10.3390/ijms241310623

**Published:** 2023-06-25

**Authors:** Elizaveta N. Mochalova, Elena A. Egorova, Kristina S. Komarova, Victoria O. Shipunova, Nelli F. Khabibullina, Petr I. Nikitin, Maxim P. Nikitin

**Affiliations:** 1 Nanobiomedicine Division, Sirius University of Science and Technology, 1 Olimpiyskiy Ave, 354340 Sirius, Russia; egorova.ea@talantiuspeh.ru (E.A.E.); viktoriya.shipunova@phystech.edu (V.O.S.); 2 Moscow Institute of Physics and Technology, 1A Kerchenskaya St., 117303 Moscow, Russia; komarovakristina1993@gmail.com (K.S.K.); khabibullina.nf@mipt.ru (N.F.K.); 3 Prokhorov General Physics Institute of the Russian Academy of Sciences, 38 Vavilov St., 119991 Moscow, Russia; nikitin@kapella.gpi.ru; 4 Shemyakin-Ovchinnikov Institute of Bioorganic Chemistry, Russian Academy of Sciences, 16/10 Miklukho-Maklaya St., 117997 Moscow, Russia

**Keywords:** liposomes, doxorubicin, clodronic acid, magnetic nanoparticles, intralipid, blood circulation time, pharmacokinetics, MPS clearance, macrophages, red blood cells

## Abstract

Recent developments in the field of nanomedicine have introduced a wide variety of nanomaterials that are capable of recognizing and killing tumor cells with increased specificity. A major limitation preventing the widespread introduction of nanomaterials into the clinical setting is their fast clearance from the bloodstream via the mononuclear phagocyte system (MPS). One of the most promising methods used to overcome this limitation is the MPS-cytoblockade, which forces the MPS to intensify the clearance of erythrocytes by injecting allogeneic anti-erythrocyte antibodies and, thus, significantly prolongs the circulation of nanoagents in the blood. However, on the way to the clinical application of this approach, the question arises whether the induced suppression of macrophage phagocytosis via the MPS-cytoblockade could pose health risks. Here, we show that highly cytotoxic doxorubicin- or clodronate-loaded liposomes, which are widely used for cancer therapy and biomedical research, induce a similar increase in the nanoparticle blood circulation half-life in mice as the MPS-cytoblockade, which only gently and temporarily saturates the macrophages with the organism’s own erythrocytes. This result suggests that from the point of view of in vivo macrophage suppression, the MPS-cytoblockade should be less detrimental than the liposomal anti-cancer drugs that are already approved for clinical application while allowing for the substantial improvement in the nanoagent effectiveness.

## 1. Introduction

Nanomaterials offer tremendous potential for numerous biomedical applications such as biosensing [1], transfection [2], bioimaging [3], etc. Furthermore, nanomaterials are of primary interest as an alternative method for cancer therapy [4]. Conventional methods for cancer treatment (e.g., chemotherapy, radiation therapy) exhibit low efficacy in discriminating between cancer and normal cells, which contributes to their low specificity and many severe side effects [5,6,7,8], whereas nanoparticles can act as drug carriers and accumulate mainly in the tumor, e.g., due to the recognition of biomarkers on the surface of tumor cells or in the tumor microenvironment [9,10,11,12,13,14].

However, despite the fact that a number of nanomaterials were already approved for clinical use [15,16], their wider introduction into clinical practice is limited by their rapid elimination from the bloodstream via the mononuclear phagocyte system (MPS). Thus, most of the administered dose accumulates in the liver and spleen, bypassing the tumor or other target organs and tissues [17,18].

Previously, several strategies were proposed to decrease the MPS uptake of nanodrugs, for example, by changing their surface properties [19,20]. This was achieved through modification using “stealth” coatings [21], the display of dysopsonins [22], or self-peptides [23,24], as well as by using non-spherical particles [25]. Despite being effective, these approaches can be limiting in the case of smart and hierarchical nanomaterials. Altered surface properties or architecture may hinder other intended functionalities such as targeted delivery [26], surface transformations in logic-gated systems [27], as well as modulate essential interactions such as cellular uptake [28], etc. At the same time, several reports show an immune or inflammatory response to these materials [29,30].

Alternatively, the MPS itself can be affected to achieve the same goal. This approach implies a transient decrease in the phagocytic activity of the MPS cells due to the uptake of various blocking agents [20]. This prevents subsequently administered therapeutic or diagnostic particles from rapidly being eliminated from the bloodstream. The direct influence on the MPS is realized through the use of blocking agents that induce macrophage death [31,32]. Notable examples include pre-treatment with liposomal clodronate or gadolinium salts. However, liposomal clodronate injected intravenously (i.v.) was reported to profoundly deplete macrophages in the liver (Kupffer cells), spleen, and bone marrow for excessive periods of time, and was found to be highly dose-dependent [32]. Treatment with gadolinium salts induces similar negative effects to liposomal clodronate with respect to elevated toxicity [31,33]. Additionally, there are blocking agents that do not exhibit a capacity for macrophage death but can also manipulate the MPS. For example, intralipid, as well as SMOFlipid (soy oil, medium-chain triglyceride, olive, and fish-oil-based lipid emulsion), nutritional supplements known to be taken up by Kupffer cells in the liver, were reported to act as an inhibitor for their phagocytic activity [17,34]. However, the safety of both formulations raised concerns [35,36]. Another approach is the use of “decoy” nanoparticles that block the MPS, luring the MPS cells away from the intended nanodrug, and thus, prolonging its circulation time [37,38]. The limitations of this approach include a profound danger of dose-related toxicity and related tissue damage.

There are also indirect ways to affect the MPS that allow for the avoidance of common complications related to induced MPS cell death, in particular, an impaired immune system. One example is cell hitchhiking, most prominently of erythrocytes, or red blood cells (RBCs) [39,40,41]. Due to their inherently prolonged circulation, RBCs act as long-circulating carriers for drug-loaded nanoparticles modified in a way to be absorbed onto the RBC surface. When injected intravascularly, these modified RBCs exhibit significantly longer circulation times for the corresponding nanoparticles as opposed to the nanoparticles alone. However, cell hitchhiking is not a trivial procedure, but requires considerable effort, and cannot be implemented for an arbitrary nanomaterial.

A different approach for the MPS blockade based on macrophage saturation with antibody-sensitized RBCs is the MPS-cytoblockade [4,42]. Antibodies are injected i.v. to bind to erythrocytes and, thus, mark them for phagocytosis. This forced clearance of erythrocytes was shown to be highly effective in the prolongation of nanoparticle circulation in the bloodstream. Additionally, this method demonstrated no considerable toxicity in vivo. Unlike previously described methods, this approach has a mild and temporal effect on the MPS and does not require extensive manipulation of erythrocytes or elaboration of nanoparticle designs. However, the question of whether the saturation of macrophages with erythrocytes can inhibit the performance of the immune system, leading to dangerous side effects, remains open.

In this study, we demonstrate that commonly used anti-cancer doxorubicin-loaded liposomes, known to induce macrophage death, induce the immune system suppression to the same extent as the MPS-cytoblockade, which, on the contrary, acts gently on macrophages by temporarily saturating them with the organism’s erythrocytes. For a high-precision side-by-side comparison of the level of immune system suppression, we injected magnetic nanoparticles 24 and 12 h after administering the Dox-loaded liposomes and anti-erythrocyte antibodies, respectively. Next, we measured the magnetic nanoparticle blood circulation half-life using a high-throughput magnetic particle quantification (MPQ) technique. This method allowed for a comprehensive analysis of the removal of nanomaterials from the bloodstream in a kinetics regimen with a time resolution of about 2 s via a non-invasive manner. Moreover, by using this approach, we evaluated the efficacy of prolonging nanoparticle circulation after the introduction of intralipid/SMOFlipid, and clodronate-loaded liposomes. The obtained results indicate that the MPS-cytoblockade has a high potential for safe use in prolonging therapeutic nanomaterial circulation due to its transient effect on the immune system and the high efficiency of the method.

## 2. Results and Discussion

### 2.1. Synthesis and Characterization of Liposomes

**Figure 1:**
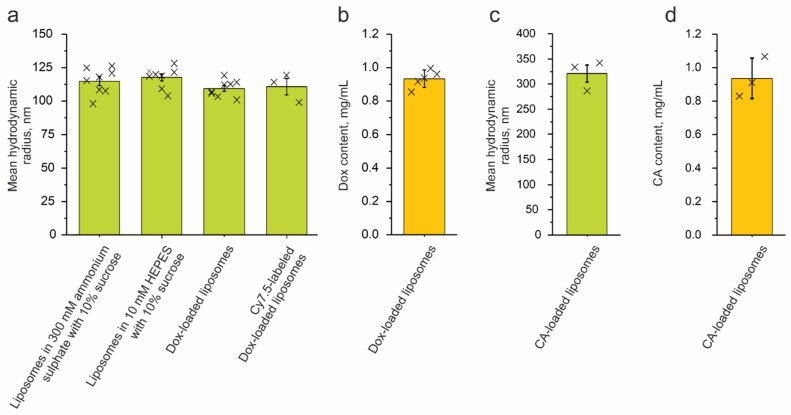
Characterization of the liposomes. (**a,c**) Mean hydrodynamic radii for different stages of synthesis, loading, and labeling. (**b,d**) Doxorubicin (Dox) and clodronic acid (CA) content, mg per mL of liposome suspension. The error bars indicate either the standard error of the mean (SEM) (**a**,**c**) or standard deviation (SD) (**b**,**d**) intervals.

First of all, we synthesized liposomes composed of egg phosphatidylcholine/cholesterol (70/30, mol/mol). Then, we encapsulated either doxorubicin (Dox) hydrochloride [43] or clodronic acid (CA) [44]. These two synthetic procedures were carried out differently. Dox loading was conducted on preformed liposomes fabricated via extrusion through a membrane with a 200 nm pore size. The loading was facilitated due to a transmembrane ammonium sulfate gradient, which was achieved via buffer exchange. For further experiments, a part of the Dox-loaded liposomes was also labeled with Cyanine7.5 amine (Cy7.5). According to a DLS analysis, the liposomes exhibited narrow size distributions with mean hydrodynamic radii of 115.0 ± 3.4 nm for the preformed liposomes in the ammonium sulfate, 117.8 ± 2.7 nm for the liposomes after the buffer was exchanged for 10 mM HEPES supplemented with 10% sucrose, 109.4 ± 2.2 nm for the Dox-loaded liposomes, and 110.7 ± 6.1 nm for the Cy7.5-labeled Dox-loaded liposomes (Figure 1a). The resulting liposomes also showed a high Dox encapsulation of 0.93 ± 0.05 mg/mL (Figure 1b). Furthermore, parallel Cy7.5 incorporation did not seem to affect the Dox encapsulation efficiency. On the other hand, the CA-loaded liposomes were prepared via sonication of the same lipids in the presence of dissolved CA. According to the DLS, these liposomes exhibited a larger size and a narrow size distribution, as their hydrodynamic radius was 320.8 ± 29.8 nm (Figure 1c); the mean clodronate content was found to be 0.94 ± 0.12 mg/mL (Figure 1d).

### 2.2. Synthesis and Characterization of MNPs

**Figure 2:**
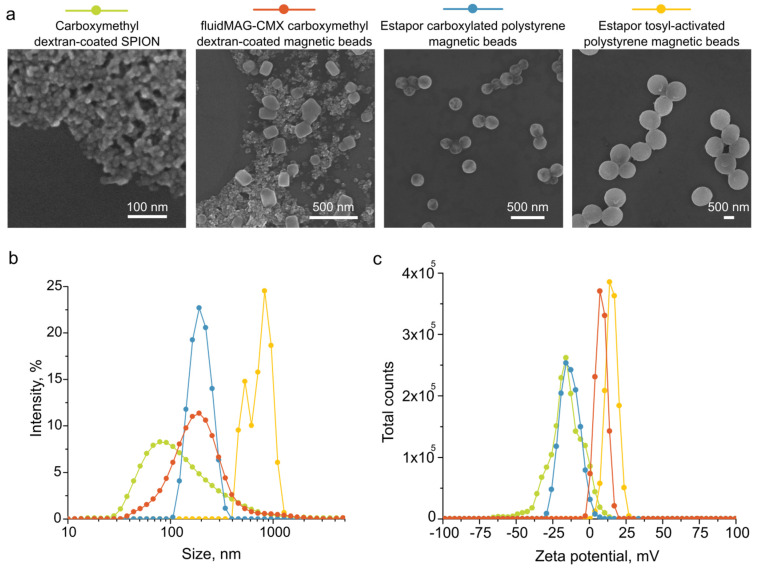
Characterization of magnetic particles. (**a**) Scanning electron microscopy images. (**b**) Diameters and (**c**) zeta potentials according to dynamic light scattering analysis.

In order to study various methods for prolonging the circulation of nanomaterials in the bloodstream, as model nanoparticles, we used commercially available Estapor carboxylated and Estapor tosyl-activated polystyrene magnetic beads, fluidMAG-CMX carboxymethyl dextran-coated magnetic beads, and superparamagnetic iron oxide nanoparticles (SPIONs) that were synthesized using a standard route of a co-precipitation of iron (II) and (III) chloride salt in alkali conditions [27] and coated with carboxymethyl dextran. To estimate the morphology of the micro- and nanoparticles, we obtained their images using a scanning electron microscope (Figure 2a). Additionally, we measured the particle sizes and zeta potentials using DLS analysis (size distributions are shown in Figure 2b; zeta potential profiles are shown in Figure 2c; and their mean values are shown in Table 1). The commercially available samples exhibited size values that were in agreement with the information provided by the manufacturers. The fluidMAG-CMX and Estapor carboxylated magnetic beads showed similar sizes (a diameter of 171.2 ± 18.0 nm versus 190.6 ± 1.7 nm); however, the fluidMAG-CMX showed a more neutral surface charge than the negatively charged Estapor carboxylated magnetic beads (6.9 ± 0.2 mV versus −12.5 ± 1.2 mV). Compared to the rest, the Estapor tosyl-activated magnetic beads had the largest average size, at 957.2 ± 150.2 nm, and a positive surface charge of 15.1 ± 0.6 mV. The SPIONs were the smallest in the row, at 97.5 ± 2.9 nm, and their zeta potential was negative (−18.3 ± 1.0 mV). This way, four model nanoparticles covered a wide range of physiochemical properties that had negatively, positively, or neutrally charged formulations in the size range from ≈100 to 1000 nm.

### 2.3. Investigation of the Prolongation of MNP Circulation in the Bloodstream

To monitor the kinetics of the circulation of MNPs in the mouse’s bloodstream, we used the magnetic particle quantification (MPQ) technique. This real-time method offers a quantitative determination of the nonlinear magnetic materials in a sample with a high sensitivity; the detection limit is 0.4 ng of nanoparticles in a 200 μL volume (or 60 zeptomoles), and the linear dynamic range is seven orders of magnitude [45]. The MPQ technique is actively used not only in the field of in vivo research for the analysis of the kinetics and biodistribution of magnetic particles [46,47], but also in the field of diagnostics for the rapid detection of toxins, hormones, extracellular vesicles, and markers of various diseases [48,49]. A sample is placed in a magnetic field generated at frequencies—f_1_ and f_2_. The device records the response at certain combinatorial frequencies of the applied field f = n·f_1_ ± m·f_2_. This principle of operation of the device allows for the measurement of the signal of nonlinear (superparamagnetic and ferromagnetic) materials, while linear (diamagnetic and paramagnetic) materials do not contribute to the final signal. In this work, the tail of the anesthetized mouse was placed in the coil of the MPQ device, then magnetic nanoparticles were injected intravenously into the retro-orbital sinus. The magnetic signal was recorded every 1.6 s until it completely dropped to the noise level.

**Figure 3:**
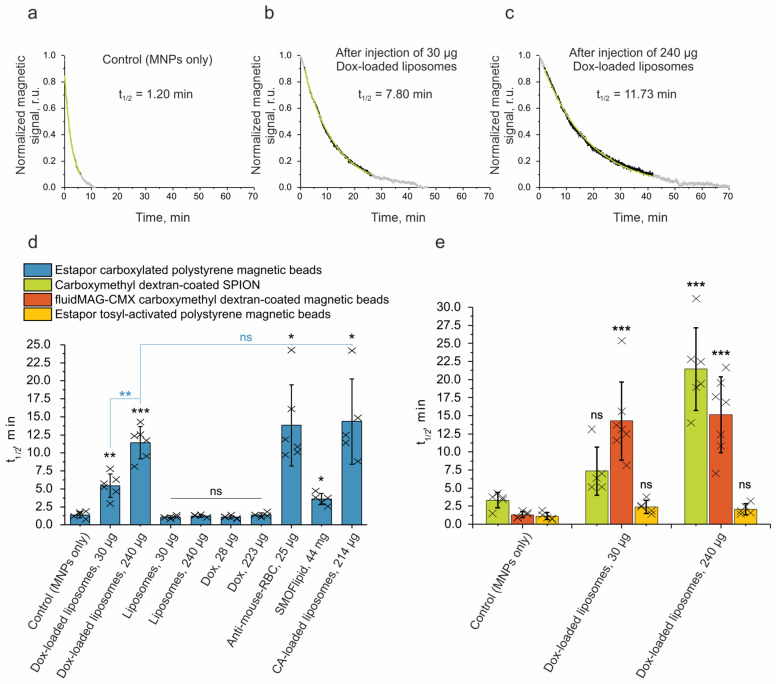
Measurements of the circulation time of MNPs in the bloodstream using the MPQ method. (**a**–**c**) Representative circulation kinetics for Estapor carboxylated polystyrene magnetic beads only and 24 h after administration of 30 and 240 µg Dox-loaded liposomes. Black dots correspond to the magnetic signal in a range of 0.9–0.1, and grey corresponds to ranges of 1.0–0.9 and 0.1–0. Green lines are the exponential fitting curves for 0.9–0.1 data points that were used to calculate the circulation half-life time (t_1/2_). (**d**) Half-life times of Estapor carboxylated polystyrene magnetic beads in a bloodstream: 24 h after injection of doxorubicin (Dox)-loaded liposomes, liposomes without Dox, and Dox; 12 h after injection of anti-mouse RBC antibodies; 1 h after injection of SMOFlipid; and 48 h post-injection of clodronic acid (CA)-loaded liposomes. (**e**) Half-life times of different magnetic nanoparticles in the bloodstream after injection of Dox-loaded liposomes. Control mice were injected with MNPs only. The number of animals was at least *n* = 5 for each group. Statistical significance for different circulation prolongation methods relative to the negative control (corresponding to MNPs only) is shown in black (**d**,**e**), while statistical significance in comparison to the 240 µg Dox-loaded liposomes is shown in blue. A *p*-value ≤ 0.05 was considered to be statistically significant (ns—*p* > 0.05; *—*p* ≤ 0.05; **—*p* ≤ 0.01; ***—*p* ≤ 0.001).

First of all, we investigated the circulation kinetics of the Estapor carboxylated polystyrene magnetic beads. A typical graph of the elimination of nanoparticles from the bloodstream (the magnetic signal, normalized to the maximum, versus time) is shown in Figure 3a. For *n* = 6 repeats, the circulation half-life was 1.38 ± 0.40 min (Figure 3d, first column).

Liposomal Dox is mainly used in cancer therapy [50]. The following two formulations were authorized for clinical use: PEGylated liposomal Dox, known by the names of Doxil^®^ (USA), Lipodox^®^ (generic Doxil), and Caelyx^®^ (Europe), and non-PEGylated liposomal Dox, known as Myocet^®^ (Canada and Europe). It was proven that these formulations reduce cardiotoxicity and hematological toxicity compared to free Dox. However, macrophages were reported to be heavily affected by the liposomal Dox [51]. The depletion of cellular subsets and impairment of the phagocytic activity of the liver macrophages was observed in rats 24 h post-administration of 5 mg/kg of liposomal Dox. Additionally, it was reported that it took 14 days to restore the phagocytic capacity after a liposomal Dox treatment. Similar trends were observed in mice [52]. The liver macrophages showed an impairment of the phagocytic function in the Doxil dose range of 2.5–20 mg/kg. At the same time, the ability for other liposomes to be cleared at 20 h post-injection of Doxil (10 mg/kg) was significantly reduced. Despite being important findings, these data were obtained as single end-point measurements for isotope-labeled liposome levels in the liver and plasma.

In our work, we used a high-throughput MPQ technique to comprehensively study the removal of nanomaterials from the bloodstream in a kinetics regimen with a time resolution of about 2 s using a non-invasive manner. We performed a high-precision side-by-side comparison of methods for prolonging the circulation of nanomaterials in the blood that do not require any modification of particles, namely, the introduction of Dox-loaded liposomes, anti-erythrocyte antibodies, intralipid/SMOFlipid, and CA-loaded liposomes.

When the Dox-loaded liposomes were administered 24 h before the injection of MNPs, the Estapor carboxylated polystyrene magnetic beads’ circulation half-life (t_1/2_) increased significantly by up to 5.43 ± 1.63 min for a 30 μg dose of Dox-loaded liposomes (by 3.9 times) and up to 11.41 ± 2.23 min for 240 μg (by 8.3 times) (Figure 3b–d). The dosage of the Dox-loaded liposomes was selected based on the available recommendations for Myocet. The administration in humans is carried out in a course of multiple injections of a 60–75 mg/m^2^ dose [53,54]. In mice, the human-equivalent dose would be ≈20–25 mg/kg [55]. In order to simulate a clinically relevant outcome, we chose the following two regimens: a single dose administration of 10 mg/kg or 240 μg per mouse (~half of the recommended dose) and 1.25 mg/kg or 30 μg per mouse (~1/20 of the recommended dose). This allowed us to study a broad Dox dose range in a window that is considered safe.

It should be noted that with the administration of the same dose of unloaded liposomes, as well as a corresponding dose of molecular doxorubicin (28 μg and 223 μg Dox), no prolongation of the circulation of magnetic nanoparticles was observed.

Next, we investigated other methods for the prolongation of nanoparticle circulation, including the MPS blockade with anti-mouse RBC antibodies, the inhibition of phagocytic activity via SMOFlipid, and macrophage depletion using CA-loaded liposomes. These inhibitors were administered i.v. in a single injection. The administration scheme was as follows: 25 μg of anti-mouse RBC antibodies, 44 mg of SMOFlipid, or 214 μg of CA-loaded liposomes (which corresponds to 200 µg of CA) were administered 12, 1, or 48 h [4,32,56] before the injection of MNPs, respectively. The effectiveness of each MPS inhibition approach was tested via Estapor carboxylated polystyrene magnetic beads (Figure 3d). As seen from the presented data, the SMOFlipid showed the lowest capacity for the prolongation of the MNP circulation compared to the other methods. However, the anti-mouse RBC antibodies and CA-loaded liposomes exhibited a similar result to the Dox-loaded liposomes (at a 240 µg dose); 13.82 ± 5.61 min and 14.34 ± 5.91 min (*p* > 0.05). Moreover, we examined the effect of the Dox-loaded liposomes on the subsequent circulation times of the various MNPs (Figure 3e). It can be seen that the Estapor tosyl-activated polystyrene magnetic beads were the least affected with prolongations of 2.2- and 1.9-fold relative to the control, for the 30 μg and 240 μg Dox-loaded liposomes, respectively. Both their large size and/or positive charge could have contributed to this outcome. At the same time, the fluidMAG-CMX and Estapor carboxylated polystyrene magnetic beads, both similar in size but slightly different in charge, showed a more effective prolongation, but a somewhat different response to the two doses of the Dox-loaded liposomes. The more neutral fluidMAG-CMX magnetic beads showed prolongations of 10.8- and 11.5-fold for the 30 μg and 240 μg Dox-loaded liposomes, while the more negative Estapor beads (Figure 3d) showed a more pronounced Dox dose dependency—prolongations of 3.9- and 8.3-fold, respectively. The SPIONs were the smallest MNPs in this study and had a negative charge. Similar to the negatively charged Estapor carboxylated polystyrene magnetic beads, the SPIONs showed a clear Dox dose dependency with prolongations of 2.2- and 6.5-fold.

### 2.4. Biodistribution Studies

**Figure 4:**
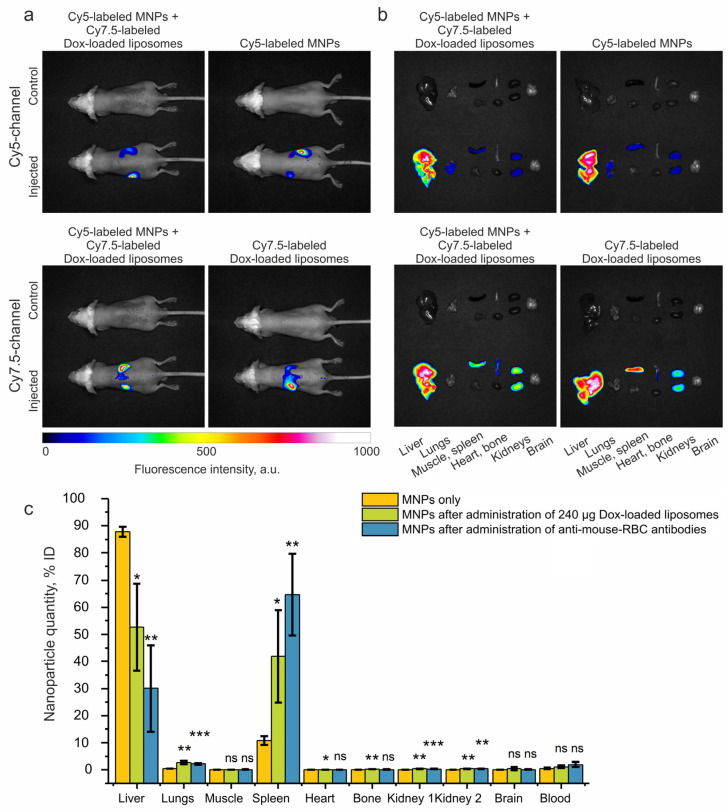
Study of the biodistribution of nanoparticles. Representative merged brightfield and Cy5 or Cy7.5 channel images revealing (**a**) in vivo and (**b**) ex vivo biodistribution of MNPs (Estapor carboxylated polystyrene magnetic beads) and Dox-loaded liposomes. Mice were injected i.v. with Cy7.5-labeled Dox-loaded liposomes 24 h before injection of Cy5-labeled magnetic nanoparticles, or injected separately with Cy7.5-labeled Dox-loaded liposomes or Cy5-labeled MNPs. Non-injected mice were used as control. (**c**) Analysis of MNP biodistribution using the MPQ method with Estapor carboxylated polystyrene magnetic beads only, and after administration of 240 µg Dox-loaded liposomes and anti-mouse RBC antibodies. The number of animals was *n* = 3 for each group. Statistical significance for different circulation prolongation methods is relative to the negative control (corresponding MNPs only). A *p*-value of ≤ 0.05 was considered to be statistically significant (ns—*p* > 0.05; *—*p* ≤ 0.05; **—*p* ≤ 0.01; ***—*p* ≤ 0.001.

Optical visualization is actively used to study the biodistribution of nanoparticles in animals [47]. To investigate the biodistribution of the MNPs and Dox-loaded liposomes both in vivo and ex vivo, we labeled the Estapor carboxylated polystyrene magnetic beads and Dox-loaded liposomes using sulfo-Cy5 amine and Cy7.5 amine, respectively. The resulting fluorescent nanoagents were i.v. injected into shaved BALB/c mice both separately and sequentially with an interval of 24 h, as described above. Then, the mice were imaged in vivo on a LumoTrace FLUO bioimaging system in Cy5 or Cy7.5 channels, and the non-injected mice were used as the autofluorescence control (Figure 4a) [57]. To demonstrate the possibility of the simultaneous detection of both fluorescent labels in the same animal, we provide a visualization of the sulfo-Cy5 amine, Cy7.5 amine solutions, and their mixtures in the Cy5 and Cy7.5 channels of the instrument (Figure S1). Additionally, the fluorescence levels were measured ex vivo in different organs and tissues (Figure 4b). The brightness and contrast in the fluorescent images were adjusted to remove the signal of the control samples and to avoid excessive signal saturation from the liver.

This qualitative biodistribution analysis revealed the accumulation of MNPs and Dox-loaded liposomes mainly in two major organs of the MPS—the liver and spleen—and a relatively low uptake of the Cy7.5-labeled Dox-loaded liposomes in the bones, and of the Cy5-labeled MNPs in the lungs. We presume that the weak fluorescent signal in the kidneys is due to the renal clearance of dye molecules unbound to the nanoparticles, as it is a known pathway for low-molecular-weight compounds [58].

In order to understand how the biodistribution of MNPs changes after the administration of Dox-loaded liposomes or anti-mouse erythrocyte antibodies, we performed a quantitative analysis of the MNP biodistribution using the MPQ method (Figure 4c, and logarithmic graph in Figure S2). The MNPs themselves showed accumulation in the liver, spleen, and lungs, which is consistent with the data obtained via optical visualization. However, after the administration of Dox-loaded liposomes or anti-mouse erythrocyte antibodies, the biodistribution significantly changed; more MNPs accumulated in the spleen and lungs, and fewer accumulated in the liver. The result shows similar patterns of MNP accumulation in the organs for both methods of circulation prolongation and correlates well with the previously obtained data [4,32].

Thus, the Dox-loaded liposomes, which were widely utilized for cancer therapy over the past 30 years [15], can also cause MPS blockade. Therefore, the mere occurrence of MPS blockade as a consequence of the introduction of new drugs should not hinder their acceptance into clinical use. This is especially relevant for the MPS-cytoblockade technique, which has immense potential for enhancing the effectiveness of targeted drug delivery utilizing nanomaterials. This may also be true for the use of Dox-loaded liposomes themselves, which are still expanding their range of applications for the treatment of various diseases [59].

## 3. Materials and Methods

### 3.1. Materials

The following reagents were used in the experiments: L-α-phosphatidylcholine from egg yolk (EPC), cholesterol, sucrose, iron (III) chloride hexahydrate, iron (II) chloride tetrahydrate, carboxymethyl dextran (CMD) sodium salt, N-(3-Dimethylaminopropyl)-N’-ethylcarbodiimide hydrochloride (EDC), N-hydroxysulfosuccinimide sodium salt (sulfo-NHS), phosphate-buffered saline (PBS) tablets (Sigma-Aldrich, St Louis, MO, USA); 2-(N-morpholino)ethanesulfonic acid (MES) monohydrate, Tris hydrochloride, EDTA, Triton X-100 (AppliChem, Darmstadt, Germany); ammonium hydroxide, nitric acid (Component-reaktiv, Moscow, Russia); sulfo-Cyanine5 amine (Cy5), Cyanine7.5 amine (Cy7.5) (Lumiprobe, Moscow, Russia); HEPES (Dia-M, Moscow, Russia), dimethyl sulfoxide (DMSO) (Chimmed, Moscow, Russia), ammonium sulfate (Helicon, Moscow, Russia); mouse antibodies against mouse red blood cells (RBC) clone 34-3C (Hycult Biotech, Uden, Netherlands); glucose for intravenous infusion (Kraspharma, Moscow, Russia); Zoletil 100 (Virbac, Carros, France); Xyla (Interchemie Werken De Adelaar Eesti AS, Püünsi, Estonia); Estapor 200-nm carboxylated and Estapor 1000 nm tosyl-activated polystyrene magnetic beads (Merck Millipore, Billerica, MA, USA); fluidMAG-CMX carboxymethyl dextran-coated magnetic beads (Chemicell, Berlin, Germany); SMOFlipid (Fresenius Kabi, Bad Homburg, Germany); doxorubicin-Teva (Pharmachemie, Haarlem, Netherlands); and clodronic acid (60 mg/mL, Bonefos^®^, Bayer, Leverkusen, North Rhine-Westphalia, Germany). The manual LiposoFast-Basic extruder, its holder, and polycarbonate membranes with a 200 nm pore size were purchased from Avestin, Ottawa, ON, Canada. NAP-5 size exclusion chromatography (SEC) columns were purchased from Cytiva, Shrewsbury, MA, USA. Milli-Q water (Merck Millipore, Billerica, MA, USA) was used for the preparation of aqueous solutions.

### 3.2. Animals

All procedures were approved by the Institutional Animal Care and Use Committee of the Shemyakin-Ovchinnikov Institute of Bioorganic Chemistry Russian Academy of Sciences according to protocol # 299 (1 January 2020–31 December 2022). Male BALB/c mice (24–26 g weight) were used in this study. Before every procedure, mice were anesthetized via intraperitoneal injection of a Zoletil 100/Xyla combination at the dose of 40/1.5 mg/kg.

### 3.3. Liposome Preparation and Loading with Doxorubicin (Dox)

Doxorubicin-loaded liposomes were prepared according to the protocol reported by A. Fritze et al. with a slight alteration [43]. A lipid mixture comprising egg phosphatidylcholine and cholesterol (70/30, mol/mol) was prepared by mixing the two corresponding lipid stock solutions in ethanol. Then, the solvent was evaporated under reduced pressure at 40 °C. The resulting dry lipid film was rehydrated with a 300 mM ammonium sulfate solution supplemented with 10% (*w*/*v*) sucrose to the final lipid concentration of 20 mM. This mixture was sonicated for 15 s at 60 °C, and then extruded 21 times through a polycarbonate membrane with a 200 nm pore size. The resulting liposomes were passed through an SEC column to replace the extra liposomal buffer for HEPES buffer (10 mM HEPES, 10% sucrose, pH 7.3). The collected liposomes were then mixed with a 10 mg/mL Dox solution (aq.) to achieve a Dox/lipid ratio of 1/3 (mol/mol), or 300 µg Dox per 1 mg of lipids. The Dox loading was conducted at 60 °C for 20 min. Next, the Dox-loaded liposomes were separated from free Dox on an SEC column with HEPES buffer as eluent.

Liposomes without Dox were prepared following the same procedure, but after the first SEC purification step, the liposomes were diluted with HEPES buffer according to the final dilution observed for Dox-loaded liposomes.

### 3.4. Liposome Preparation and Loading with Clodronic Acid (CA)

Liposomal clodronate was prepared according to the method developed by van Rooijen et al. with minor alterations [44]. The liposomes were synthesized using the lipid film hydration method. The lipid composition was as follows: EPC:Cholesterol = 70:30 (mol/mol). A solution of clodronic acid (60 mg/mL) was added to the dry lipid film at the total lipid/clodronate ratio of 1:2.67 (*w*/*w*). The vial was sonicated in an ultrasonication bath (Sonorex digitec DT 52H, Bandelin, Germany) and preheated to 50 °C for 15 min. After that, the liposomes were incubated at 4 °C overnight. To remove free clodronate and exchange buffer for PBS, the resulting liposomes were washed with PBS via centrifugation (5000× *g*, 5 min, 3 times).

### 3.5. Characterization of Liposomes

Dynamic light scattering (DLS) was used to evaluate the hydrodynamic radius of the studied liposomes. Size distributions by intensity were obtained using Photocor Compact-Z (Photocor, Moscow, Russia) standard protocols. The temperature was maintained at 23 °C during the measurements. An average of 20 runs was taken as the mean value. For the determination of doxorubicin encapsulation efficiency, the liposomes were disrupted with Triton X-100 (final concertation of 0.5%, *v*/*v*) before and after Dox inclusion. The Dox content in the samples was measured at 495 nm using a Multiskan Spectrum plate reader (Thermo Fisher Scientific, Cleveland, OH, USA), and their corresponding encapsulation efficiencies were calculated. Clodronate content in liposomal clodronate was determined through the complexation of CuSO_4_ as previously described [44]. For that purpose, an absorbance reading at λ = 236 nm was used (UV clear 96-well plates; Clariostar, BMG Labtech, Ortenberg, Germany). Obtained values were compared to a calibration curve built for known clodronate concentrations, and thus, the mean content was calculated.

### 3.6. Synthesis of Superparamagnetic Iron Oxide Nanoparticles (SPIONs)

SPIONs were synthesized using a standard route of co-precipitation of iron (II) and (III) chloride salt in alkali conditions [27] and coated with carboxymethyl dextran (CMD). CMD solution at 300 g/L in water was added to the nanoparticle suspension to obtain the final concentration of 50 g/L, followed by a 4 h incubation at 80 °C. Then, nanoparticles were washed with water three times via centrifugation at 16,000 *g* for 1–3 h.

### 3.7. Characterization of Magnetic Nanoparticles (MNPs)

Scanning electron microscopy images were obtained using MAIA3 (Tescan, Brno, Czech Republic) microscope at an accelerating voltage of 7 kV. The hydrodynamic size was measured in PBS and zeta potential was measured in 10 mM KNO_3_ using a Zetasizer Nano ZS (Malvern Instruments, Malvern, Worcestershire, UK) device.

### 3.8. Measurement of Magnetic Nanoparticle Circulation Half-Life in the Bloodstream of Mice

The tail of the anesthetized mouse was placed in the coil of the magnetic particle quantification (MPQ) device [45], then 300 μg of magnetic nanoparticles were injected i.v. into the retro-orbital sinus. The magnetic signal was recorded every 1.6 s until it completely dropped to the noise level. To determine the circulation half-life of the nanoparticles, we plotted the magnetic signal, normalized to the maximum, versus time. Next, the signal values in the range from 0.9 to 0.1 were approximated by the curve y = ae^bx^. Then, from the values of the coefficients, the half-life was calculated using the formula t_1/2_ = ln2/(−b). When investigating methods for prolonging circulation time, 30 μg and 240 μg Dox-loaded liposomes, 30 μg and 240 μg liposomes without Dox, or 28 μg and 223 μg Dox (corresponding to the amount of Dox in the loaded liposomes) were administered 24 h before the injection of MNPs. A total of 25 μg anti-mouse RBC antibodies, 44 mg SMOFlipid, or 214 μg CA-loaded liposomes were administered 12, 1, or 48 h before the injection of MNPs, respectively.

### 3.9. Labeling of Liposomes

Cyanine7.5 amine-labeled Dox-loaded liposomes were prepared for the in vivo and ex vivo imaging study. The dye was passively incorporated into the lipid bilayer. Cy7.5 amine (Cy7.5) was added to the lipid mix in ethanol (8 µg of Cy7.5 in ethanol per 1 mg of lipids). After that, the procedure was conducted as described above.

### 3.10. Labeling of 200 nm Estapor Magnetic Nanoparticles

Sulfo-Cyanine5 amine-labeled 200 nm Estapor nanoparticles were prepared for the in vivo and ex vivo imaging study. A total of 6 mg EDC and 3 mg sulfo-NHS in 40 µL of MES buffer (pH 5.0) were added to 0.3 mg 200 nm Estapor nanoparticles and incubated for 30 min under sonication. Then, 8 µg sulfo-Cyanine5 amine (Cy5) in 5% DMSO in PBS solution were added to nanoparticles and incubated overnight in a horizontal shaker. After that, 20 µL of 50 mM Tris-HCl and 5 mM EDTA (pH 8.0) in water was added, and the resulting solution was incubated for 1 h at permanent stirring. Then, nanoparticles were washed out with water five times using a magnet.

### 3.11. Biodistribution of Magnetic Nanoparticles and Liposomes

The in vivo and ex vivo optical visualizations of nanoparticle biodistribution were performed on a LumoTrace FLUO bioimaging system (Abisense, Sirius, Krasnodar region, Russia). A total of 240 μg of Cy7.5-labeled Dox-loaded liposomes were i.v. administered into the retro-orbital sinus of shaved mice 24 h before i.v. injection of 300 μg of Cy5-labeled 200 nm Estapor nanoparticles in 100 μL of 5% glucose solution, *n* = 3. The procedure was also repeated with a separate injection of liposomes and MNPs at the doses mentioned above, *n* = 3 for each type of nanoparticles. A total of 3 h after injection, the mice were imaged with 100 ms exposure. 630 nm diode and 650 nm long-pass filter or 730 nm diode and 780 nm long-pass filter was used for Cy5 and Cy7.5 channel visualizations, respectively. Non-injected mice were used as control. Additionally, fluorescence levels were measured ex vivo in the liver, lungs, muscle, spleen, heart, bone, kidneys, and brain using the same settings.

For the MPQ analysis of MNP biodistribution, the organs and tissues were extracted 2 h after nanoparticle injection and placed in the measuring coil of the MPQ detector. The integral magnetic signal from each organ was normalized to the total signal from all measured organs.

### 3.12. Statistical Analysis

Nanoparticle characterization was performed for each batch. Parameters like the size and zeta potential were obtained as mean values with a standard deviation (SD). The cumulative liposome characterization based on multiple batches is shown as mean values with the SD or standard error of the mean (SEM) depending on the experiment. All in vivo experiments were carried out as independent biological repeats; for each experiment, the number of animals in the test group (*n*) is indicated in the text. The data were presented as bar graphs showing mean values and error bars indicating the SD. Statistical analysis was performed using GraphPad Prism software version 8.0. A one-way ANOVA test was employed to study multiple data sets (or conditions). A *p*-value of ≤0.05 was considered to be statistically significant (ns—*p* > 0.05; *—*p* ≤ 0.05; **—*p* ≤ 0.01; ***—*p* ≤ 0.001; ****—*p* ≤ 0.0001).

## 4. Conclusions

Our work demonstrated that MPS-cytoblockade, which involves the forced clearance of the organism’s own erythrocytes, can increase the half-life of the nanoparticles in the bloodstream of mice to a similar extent as conventional cytotoxic doxorubicin-loaded liposomes. This suggests that the mild effect of the MPS-cytoblockade on macrophages is just as effective in inhibiting the immune system as the drugs that are already widely used in cancer therapy, which act considerably harsher, leading to macrophage death. These findings indicate that the MPS-cytoblockade has a significant potential for its safe implementation in clinical use.

Additionally, the MPS-cytoblockade has an important advantage over other blocking agents in the form of lipid-based nanoparticles or oil-based emulsions, such as Dox-loaded liposomes, CA-loaded liposomes, and intralipid/SMOFlipid, which were studied in this work. The method only intensifies the natural route of erythrocyte recycling from the body, which is approximately 2.5% of the total erythrocytes daily in a healthy mouse organism.

The MPS-cytoblockade can be a potent addition to the existing advanced methods of cancer therapy utilizing nanomaterials since the method (1) significantly improves the effectiveness of nanodrugs while maintaining their dose or (2) significantly reduces the nanodrug dose, and therefore, reduces their dangerous side effects while maintaining the effectiveness of the nanodrugs. The technique can also significantly expand the boundaries of nanoparticle applications not only in the field of oncotherapy, but also in the treatment of other numerous diseases that require targeted drug delivery.

## Figures and Tables

**Table 1:** Characterization of magnetic particles.

Nanoparticles	Manufacturer	Mean Hydrodynamic Diameter ± SD, nm	Zeta Potential ± SD, mV
Carboxymethyl dextran-coated SPIONs	This work	97.5 ± 2.9	−18.3 ± 1.0
fluidMAG-CMX carboxymethyl dextran-coated magnetic beads	Chemicell, Germany	171.2 ± 18.0	6.9 ± 0.2
Estapor carboxylated polystyrene magnetic beads	Merck Millipore, USA	190.6 ± 1.7	−12.5 ± 1.2
Estapor tosyl-activated polystyrene magnetic beads	Merck Millipore, USA	957.2 ± 150.2	15.1 ± 0.6

## Data Availability

All data are presented within the manuscript.

## Supplementary Materials

The following supporting information can be downloaded at https://www.mdpi.com/article/10.3390/ijms241310623/s1.
