# Supplementary material for: Comparative Study of Nanoparticle Blood Circulation after Forced Clearance of Own Erythrocytes (Mononuclear Phagocyte System-Cytoblockade) or Administration of Cytotoxic Doxorubicin- or Clodronate-Loaded Liposomes
Source: Int J Mol Sci. 2023 Jun 25;24(13):10623. doi: 10.3390/ijms241310623 (PMC10341719; doi:10.3390/ijms241310623)
Supplement: Supplementary file 1 [file ijms-24-10623-s001.zip › ijms-2430269-supplementary.pdf]

Supplementary Material

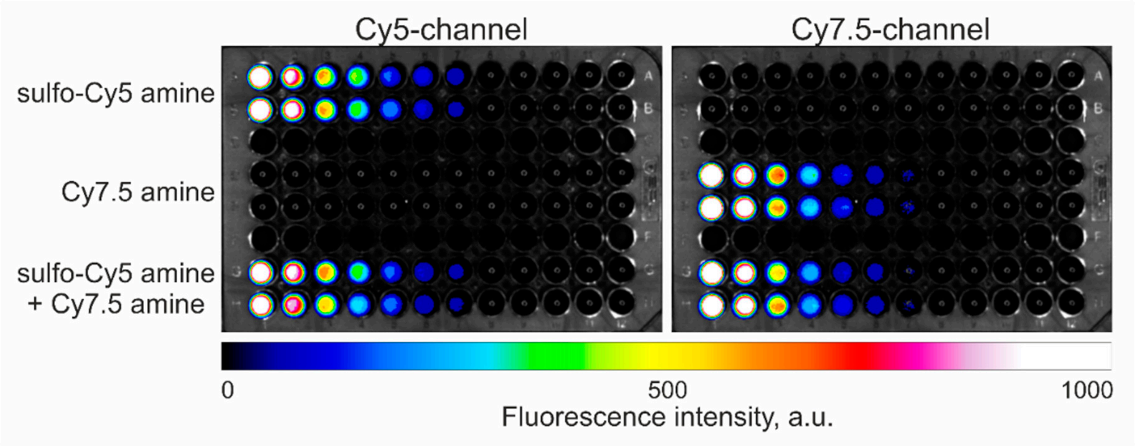

Figure S1. Visualization of the sulfo-Cy5 amine and Cy7.5 amine solutions in Cy5 and Cy7.5 channels of a LumoTrace FLUO bioimaging system used in the study. The dyes and their mixture were dissolved in 5% DMSO to a final concentration of each dye of 3.33 mg/L and were placed in the first wells (column 1, far left). In all subsequent wells (columns 2-11), the dye concentrations consistently decreased by a factor of 2. In the last wells (column 12, far right) is a 5% DMSO solution without dyes. Each panel was made in two repetitions from top to bottom for: sulfo-Cy5 amine (rows A and B), Cy7.5 amine (rows D and E), and their mixture (rows G and H). The plate was imaged with a 50-ms exposure; an excitation 630-nm diode and 650-nm long-pass filter or 730-nm diode and 780-nm long-pass filter were used for Cy5- and Cy7.5-channel visualizations, respectively.

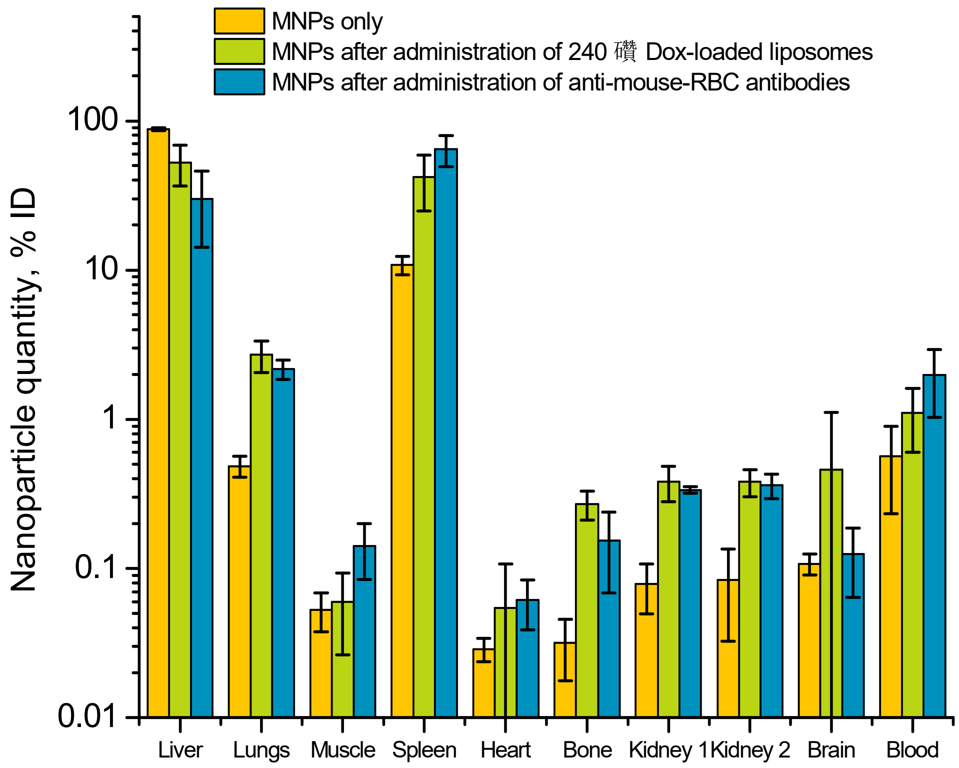

Figure S2. Analysis of MNP biodistribution using the MPQ method: Estapor carboxylated polystyrene magnetic beads only, and after administration of 240 µg Dox-loaded liposomes, and anti-mouse-RBC antibodies. The number of independent animals was n = 3 for each group.
